# Calcium Dynamics of the Ventrolateral Preoptic GABAergic Neurons during Spontaneous Sleep-Waking and in Response to Homeostatic Sleep Demands

**DOI:** 10.3390/ijms24098311

**Published:** 2023-05-05

**Authors:** Andrey Kostin, Md. Aftab Alam, Anton Saevskiy, Chenyi Yang, Peyman Golshani, Md. Noor Alam

**Affiliations:** 1 Research Service (151A3), Veterans Affairs Greater Los Angeles Healthcare System, Sepulveda, Los Angeles, CA 91343, USA; andrey.kostin@usa.com (A.K.); aftabalam@ucla.edu (M.A.A.); pgolshani@mednet.ucla.edu (P.G.); 2 Department of Psychiatry, University of California, Los Angeles, CA 90095, USA; 3 Scientific Research and Technology Center for Neurotechnology, Southern Federal University, 344006 Rostov-on-Don, Russia; saevskiy@sfedu.ru; 4 Department of Anatomy and Neurobiology, School of Medicine, University of California, Irvine, CA 92697, USA; celinaycy@gmail.com; 5 Department of Neurology, David Geffen School of Medicine, University of California, Los Angeles, CA 90095, USA; 6 Department of Medicine, David Geffen School of Medicine, University of California, Los Angeles, CA 90095, USA

**Keywords:** sleep, hypothalamus, ventrolateral preoptic area, GABA, GCaMP6, sleep homeostasis, calcium imaging

## Abstract

The ventrolateral preoptic area (VLPO) contains GABAergic sleep-active neurons. However, the extent to which these neurons are involved in expressing spontaneous sleep and homeostatic sleep regulatory demands is not fully understood. We used calcium (Ca^2+^) imaging to characterize the activity dynamics of VLPO neurons, especially those expressing the vesicular GABA transporter (VGAT) across spontaneous sleep-waking and in response to homeostatic sleep demands. The VLPOs of wild-type and VGAT-Cre mice were transfected with GCaMP6, and the Ca^2+^ fluorescence of unidentified (UNID) and VGAT cells was recorded during spontaneous sleep-waking and 3 h of sleep deprivation (SD) followed by 1 h of recovery sleep. Although both VGAT and UNID neurons exhibited heterogeneous Ca^2+^ fluorescence across sleep-waking, the majority of VLPO neurons displayed increased activity during nonREM/REM (VGAT, 120/303; UNID, 39/106) and REM sleep (VGAT, 32/303; UNID, 19/106). Compared to the baseline waking, VLPO sleep-active neurons (*n* = 91) exhibited higher activity with increasing SD that remained elevated during the recovery period. These neurons also exhibited increased Ca^2+^ fluorescence during nonREM sleep, marked by increased slow-wave activity and REM sleep during recovery after SD. These findings support the notion that VLPO sleep-active neurons, including GABAergic neurons, are components of neuronal circuitry that mediate spontaneous sleep and homeostatic responses to sustained wakefulness.

## 1. Introduction

Multiple lines of studies support the notion that the ventrolateral preoptic area (VLPO) is involved in the regulation of a broad repertoire of vital functions, including thermoregulation, sexual behavior, and sleep [1,2,3,4,5,6] VLPO lesions result in significant sleep fragmentation and insomnia or decreased sleep [7]. The VLPO contains a high density of sleep-active neurons or neurons that exhibit increasing discharge from waking → non-rapid eye movement (nonREM) sleep transition → stable nonREM/REM sleep, indicating that these neurons are involved in the initiation/maintenance of sleep [8,9]. VLPO neurons also exhibit sleep-associated fos-expression (Fos-IR), which is a marker of neuronal activation [10,11] Using the DREADD approach, we recently found that even a unilateral and selective activation of VLPO neurons projecting to the hypocretin field of the perifornical–lateral hypothalamic area (PF-LHA) was sufficient to induce sleep during the dark phase when rats are typically awake as well as during exposure to a novel environment, which causes acute arousal during the light phase [12]. Most of the neurons in the VLPO that exhibit sleep-associated Fos-IR contain inhibitory neurotransmitters, gamma-aminobutyric acid (GABA), and/or galanin [13,14]. Recent studies have focused on the contributions of VLPO’s specific neuronal groups in sleep-wake regulation [15,16,17,18]. While some inconsistencies were observed, depending on the experimental paradigm, these studies generally found that selective activation of the VLPO GABAergic or galaninergic neurons increased nonREM sleep [16,17,19,20,21]. Immunohistochemical studies suggest that while most of the sleep-active neurons are GABAergic, most of the GABAergic neurons in the VLPO are not sleep-active [10,14] Especially in mice, these sleep-active neurons are diffusely distributed in the VLPO core and its adjoining areas or an extended VLPO and are mixed with wake-active neurons [16,18,22].

Some evidence also suggests that VLPO neurons are involved in building the homeostatic sleep drive during sleep deprivation (SD) and its dissipation during recovery sleep (RS). VLPO sleep-active neurons, but not wake and state-indifferent neurons, exhibit increased discharge with increasing durations of SD that results in the progressive buildup of homeostatic sleep pressure, and declines with time paralleling to the declining EEG slow-wave activity (SWA) during the recovery period in rats [8]. A higher number of VLPO neurons exhibiting Fos-IR have also been reported in rats sacrificed at the end of 2 h of SD and after the recovery period [23]. Although mice have become the animal model of choice in sleep research due to the increasing availability of transgenic models, the activity profiles of VLPO GABAergic neurons across sleep-waking and with the waxing and waning of homeostatic sleep drive in mice are not known.

The VLPO contains a diverse and intermingled population of neurons as regards their neurotransmitter phenotypes and anatomical and functional profiles, including their roles in sleep-wake control. Until recently, most data on sleep-wake activity profiles of VLPO neurons came from electrophysiological and immunohistochemical studies, both of which have significant limitations. In recent years, however, a newly developed deep-brain imaging technique, which detects shifts in calcium dynamics in individual neurons as a readout of the action potentials in freely behaving animals, has gained popularity due to many advantages [24,25,26]. Using this approach, unlike extracellular or juxtacellular electrophysiological recording or immunohistochemical studies, the real-time dynamics of a large number of individual neurons or specific neuronal groups identified genetically or anatomically across the neuraxis can be simultaneously recorded for many days or weeks in response to a variety of tasks or behaviors, including sleep-waking in freely behaving animals [27,28,29].

In this study, we characterized the activity dynamics of VLPO neurons in general and VLPO’s GABAergic neurons in particular during spontaneous sleep-waking and across the waxing and waning of homeostatic sleep pressure using cellular-resolution Ca^2+^ imaging in freely behaving mice. Using a genetically encoded Ca^2+^ fluorescent sensor, we show that the activity of VLPO neurons, including neurons that express the gene for vesicular GABA transporter (VGAT), a reliable marker of GABAergic neurons, are strongly modulated by sleep-waking states and homeostatic sleep regulatory demands. While these neurons exhibit diverse activity profiles in relation to the sleep-waking state, the majority of VLPO neurons, including GABAergic neurons, exhibit increased Ca^2+^ activity during nonREM and or REM sleep. These sleep-active neurons also seem to be involved in homeostatic sleep control, as indicated by their increased Ca^2+^ activity with increasing durations of sleep deprivation (compared to the baseline waking activity) and during rebound nonREM sleep with increased SWA and REM sleep during the recovery period. These findings are consistent with the role of VLPO neurons in sleep control.

## 2. Results

### 2.1. Location of the GRIN Lenses and the Transfected VLPO Neurons

**Figure 1:**
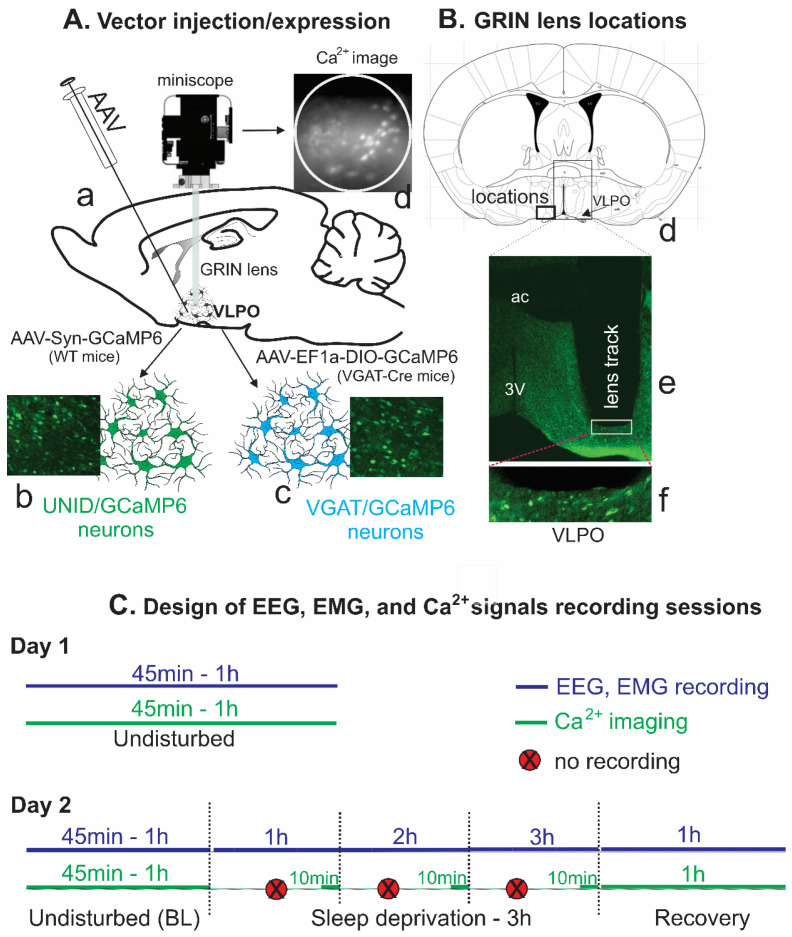
Schematic of the experimental procedures/designs used for Ca^2+^ imaging of VLPO GABAergic and UNID neurons. (**A**) Schematic representation of viral vector injections into the VLPO, implantation of the GRIN lens, and placement of miniscope used for Ca^2+^ imaging (a); photomicrographs of representative histological sections showing transfection of UNID neurons in WT mice (b) and VGAT neurons in VGAT-Cre mice (c). (**B**) Schematic representation of the locations of GRIN lenses (d). Although AP, dorso-ventral, and medial-lateral locations of viral vector injections and implanted GRIN lens varied by 50–100 μm, they were mostly confined to the boxed area (shown in one plane) that included VLPO core and extended VLPO. Photomicrograph of a representative histological section through the VLPO region showing the location of the GRIN lens atop of the VGAT neurons transfected with GCaMP6 (e), and a higher magnification image of the marked area showing the tip of the GRIN lens and VGAT/GCaMP6 neurons within the focal plane of the GRIN lens (f). The locations of the GCaMP injection sites were broadly comparable in both WT and VGAT-Cre mice. Thus, the majority of UNID and VGAT neurons were likely recorded largely from comparable and overlapping areas of the VLPO. (**C**) Schematic of the experimental design and the recording sessions. ac, anterior commissure, 3 V, third ventricle.

A representative photograph of the GRIN lens atop the cluster of VLPO VGAT neurons transfected with GCaMP6 is shown in Figure 1. The tips of the GRIN lenses mostly ended on the top or in the VLPO, and both the transfected VGAT neurons in VGAT-Cre mice and unidentified (UNID) neurons in WT mice were within the working field of the GRIN lenses. In all mice (VGAT-Cre, *n* = 7; WT, *n* = 3), the transfected VGAT and UNID neurons were located predominantly within the dorsal to the ventral extent of the LPO and adjacent areas (Figure 1), which are known to contain sleep-active neurons [8,15,16]. Based on the cytoplasmic expression of GCaMP6 within VGAT and UNID neurons, we were able to monitor Ca^2+^ dynamics of a multitude of neurons across many spontaneous sleep-waking cycles and in response to the waxing and waning of homeostatic sleep pressure during SD and recovery sleep (RS). All neurons were imaged at a single focal plane of the miniscope that yielded the sharpest images.

### 2.2. Ca^2+^ Dynamics of the VLPO VGAT Neurons across Spontaneous Sleep-Waking

**Figure 2:**
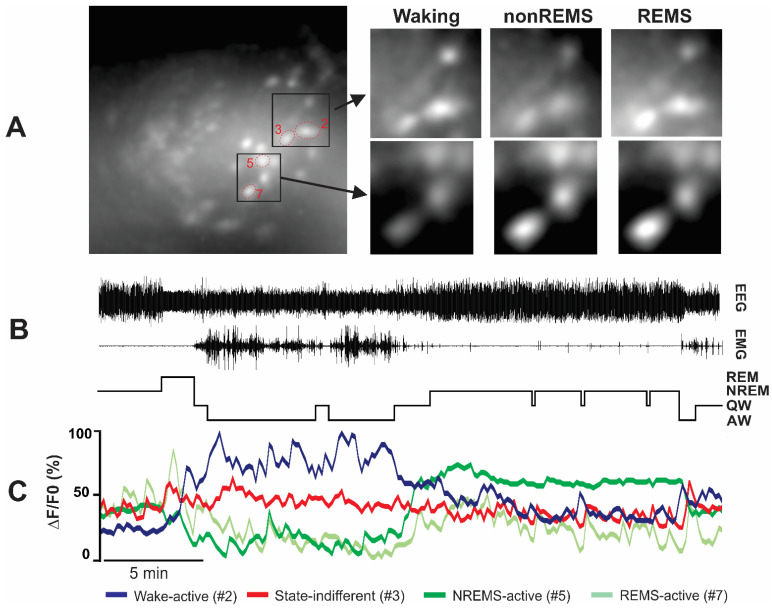
Activity dynamics of VLPO VGAT neurons across spontaneous sleep-waking. as measured by levels of GCaMP6 fluorescence. (**A**) Representative photomicrographs showing VGAT/GCaMP6+ neurons as visualized by and within the focal plane of the miniscope and changes in fluorescence levels during waking, nonREM, and REM sleep of the marked neurons. (**B**) An example of continuous EEG and EMG tracings along with the hypnogram showing behavioral states of the mice. (**C**) Continuous tracings showing changes in fluorescence levels (normalized ΔF/F_0_) of the marked neurons in relation to sleep-waking state changes as shown in (**B**). VLPO neurons were further classified based on their nonREMS/AW, REMS/AW, and nonREMS/REMS ΔF/F_0_ ratios. VLPO neurons exhibited diverse activity patterns and high variability in their Ca^2+^ dynamics across sleep-waking, which can be seen in (**C**).

**Figure 3:**
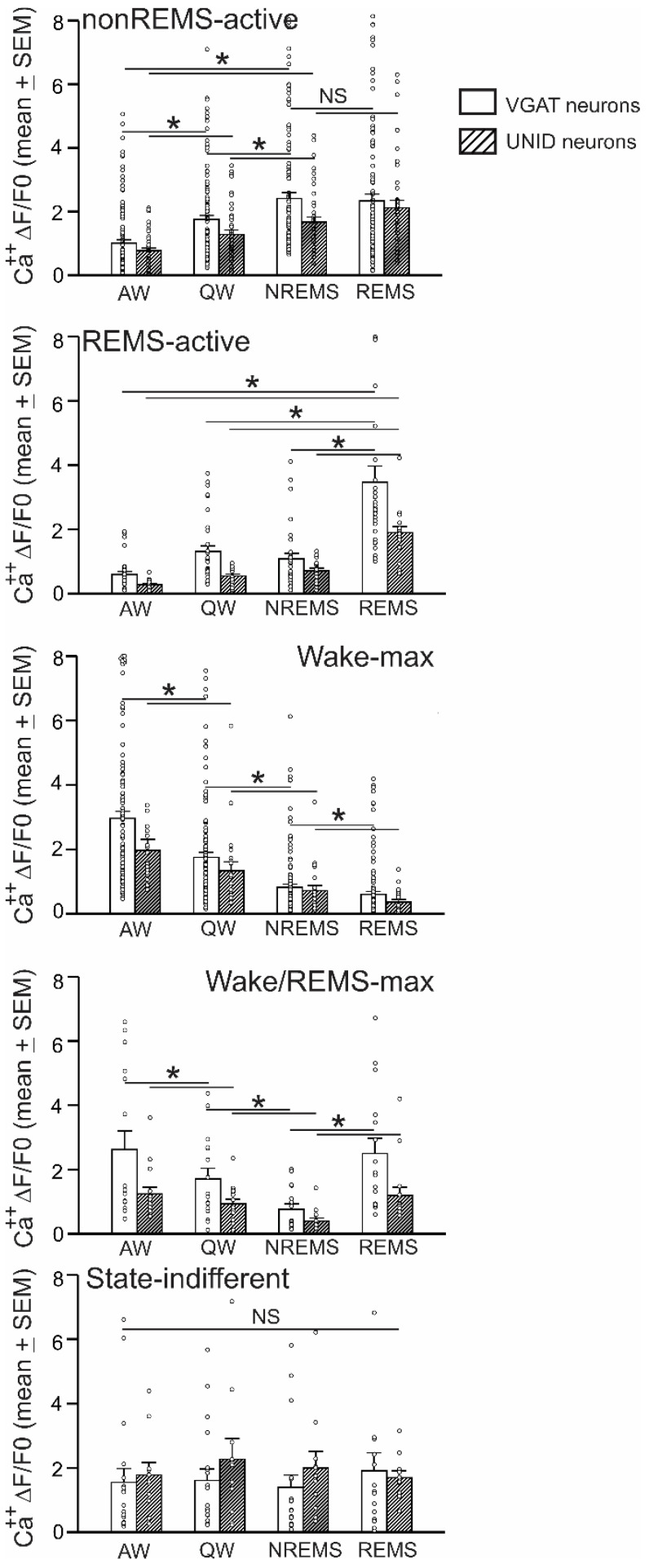
Various sleep-waking neuronal phenotypes identified in the VLPO based on their Ca^2+^ dynamics. Ca^2+^ ΔF/F_0_ (mean ± SEM; bars) of different neuronal groups identified in the VLPO during AW, QW, nonREM sleep, and REM sleep in WT (106 UNID neurons from 3 mice) and VGAT-Cre mice (303 VGAT neurons from 7 mice). Circles represent activity dynamics of individual neurons in each group. Both UNID and VGAT group contained similar sleep-waking state-related neuronal phenotypes. *, *p* < 0.05 level of significance, Friedman RM ANOVA on Ranks followed by Student–Newman–Keuls test); NS, not significant.

The Ca^2+^ dynamics of 303 VGAT neurons were recorded across at least three spontaneous sleep-waking cycles from seven VGAT-Cre mice. The imaged VGAT neurons exhibited high variability in Ca^2+^ dynamics across sleep-waking states and within states. Overall, the recorded VGAT neurons exhibited comparable ΔF/F_0_activity (Friedman RM ANOVA on ranks; *p* = 0.06) during AW (1.83 ± 0.11, range 0.01–9.49), QW (1.70 ± 0.08, range 0.11–7.52), nonREM sleep (1.51 ± 0.10, range 0.01–9.04), and REM sleep (1.77 ± 0.13, range 0.01–10.82). However, based on the nonREMS/AW, REMS/AW, and nonREMS/REMS ΔF/F_0_ ratios, these neurons were identified as functionally heterogeneous and broadly classified into three major categories: sleep-active, wake-active, and state-indifferent neurons (Table 1). The ΔF/F_0_activity profiles of various individual neurons and their mean ΔF/F_0_activity as a group across sleep-waking states are shown in Figure 2 and Figure 3.

#### 2.2.1. Sleep-Active Neurons

This group represented 50% (152 of 303 neurons) of all the recorded VGAT neurons in this study. These neurons exhibited higher ΔF/F_0_ activity during one or both phases of sleep compared to waking. Based on the specificity of ΔF/F_0_ activity during nonREM or REM sleep, these neurons were further identified as nonREM sleep-active and REM sleep-active neurons.

*a. nonREM sleep-active neurons:* These neurons constituted the majority of the sleep-active neurons (120 of 152; 79%). The spontaneous ΔF/F_0_ activity of a typical nonREM sleep-active neuron across the spontaneous sleep-waking cycle, as well as the mean ΔF/F_0_ activity of these neurons as a group along with the individual activity profiles of all the recorded neurons during waking, nonREM sleep, and REM sleep are shown in Figure 2 and Figure 3. There were significant effects of sleep-waking state changes on the ΔF/F_0_ activity of these neurons (Chi-square = 194.82; *p* < 0.001; Friedman RM ANOVA on Ranks followed by Student–Newman–Keuls method). These neurons, as a group, exhibited the lowest ΔF/F_0_ activity during AW, which increased significantly during QW (*p* < 0.05). During nonREM sleep, the mean ΔF/F_0_ activity of these neurons increased further compared to AW or QW (*p* < 0.05,) with all the neurons exhibiting similar patterns (Figure 3). As a group, these neurons exhibited comparable ΔF/F_0_ activity during nonREM and REM sleep. However, based on the fluorescence activity during REM sleep, these neurons were further classified into two sub-categories.

(i) nonREMS/REMS-max neurons: These neurons exhibited the maximum ΔF/F_0_ activity during both nonREM and REM sleep and constituted the majority of the nonREM sleep-active neurons (96 of 120; 80%). The ΔF/F_0_ activity of these neurons changed significantly in relation to the sleep-waking state changes (Chi-square = 193.71; *p* < 0.001). These neurons, as a group, exhibited the lowest ΔF/F_0_ activity during AW, which increased significantly during QW (1.04 ± 0.12 versus 1.86 ± 0.15, *p* < 0.05). During nonREM sleep, the mean ΔF/F_0_ activity of these neurons increased further compared to AW (2.54 ± 0.22 versus 1.04 ± 0.12, *p* < 0.05) or QW (2.54 ± 0.22 versus 1.86 ± 0.15, *p* < 0.05), with most neurons exhibiting a similar pattern. However, these neurons, as a group, exhibited comparable Ca^2+^ dynamics during nonREM and REM sleep (2.54 ± 0.22 versus 2.76 ± 0.25, NS). The mean REMS/nonREMS ΔF/F_0_ activity ratio was 1.09 ± 0.01, and the majority of these neurons exhibited <25% changes in REM sleep compared to nonREM sleep.

Based on the percentage increase in ΔF/F_0_ activity during nonREM sleep compared to AW, eleven neurons were identified as weak (25–50%), nine as moderate (>50–100%), and 76 as strong (>100%) nonREMS/REMS-max neurons.

(ii) nonREMS-max neurons: Of 120 nonREM sleep-active VGAT neurons, 24 (20%) neurons exhibited the maximum ΔF/F_0_ activity during nonREM sleep compared to both REM sleep and waking. Sleep-waking state changes significantly affected the ΔF/F_0_ activity of these neurons (Chi-square = 53.85; *p* < 0.001; Friedman RM ANOVA on Ranks followed by Student–Newman–Keuls method). These neurons, as a group, exhibited the highest ΔF/F_0_ activity during nonREM sleep compared to AW (1.90 ± 0.28 versus 0.87 ± 0.19, *p* < 0.05), QW (1.90 ± 0.28 versus 1.33 ± 0.24; *p* < 0.05), and REM sleep (1.90 ± 0.28 versus 0.61 ± 0.14; *p* < 0.05), with all the neurons exhibiting a similar pattern.

Except for the significant difference in ΔF/F_0_ activity in REM sleep (2.76 ± 0.25 versus 0.61 ± 0.14, *p* < 0.05; Kruskal–Wallis one-way ANOVA on Ranks followed by Dunn’s method), nonREMS/REMS-max and nonREMS-max neurons exhibited comparable ΔF/F_0_ activity in AW, QW, and nonREM sleep (*p* > 0.05). Therefore, it is likely that the two neuronal groups are variations of the same neuronal phenotype and contribute similarly to or share one or more of the components of the sleep regulatory function.

*b. REM sleep-active neurons:* Of the 152 sleep-active neurons, 32 (21%) were categorized as REM sleep-active neurons. The continuous ΔF/F_0_ dynamics of a typical REM sleep-active neuron across the sleep-waking cycle and the ΔF/F_0_ activity of all the recorded individual neurons and their mean ΔF/F_0_ activity in AW, QW, nonREM sleep, and REM sleep are shown in Figure 2 and Figure 3. There were significant effects of sleep-waking state changes on the ΔF/F_0_ activity of these neurons (Chi-square = 71.44; *p* < 0.001). These neurons, as a group, exhibited the highest ΔF/F_0_ activity during REM sleep compared to AW (3.47 ± 0.50 versus 0.60 ± 0.09, *p* < 0.05), QW (3.47 ± 0.50 versus 131 ± 0.18; *p* < 0.05), and nonREM sleep (3.47 ± 0.50 versus 1.08 ± 0.17; *p* < 0.05), with every neuron exhibiting a similar pattern.

Based on the percentage increase in ΔF/F_0_ activity during REM sleep compared to AW (REMS/AW ratio) and nonREM sleep (REMS/nonREMS ratio), except for one, all of the recorded neurons were identified as strongly REM sleep-active (>100% increase).

#### 2.2.2. Wake-Active Neurons

This group represented 44% (132 of 303 neurons) of all the imaged VGAT neurons in the VLPO. The spontaneous ΔF/F_0_ activity of a typical wake-active neuron across the spontaneous sleep-waking cycle, as well as the mean and individual ΔF/F_0_ activity profiles of all the recorded wake-active neurons across sleep-waking states, are shown in Figure 2 and Figure 3. There were significant effects of sleep-waking state changes on the ΔF/F_0_ activity of these neurons (Chi-square = 272.41; *p* < 0.001). These neurons, as a group, exhibited the highest ΔF/F_0_ activity during AW, which declined significantly during QW (2.92 ± 0.20 versus 1.74 ± 0.14; *p* < 0.05) and decreased further to a minimal level during nonREM sleep (1.74 ± 0.14 versus 0.81 ± 0.09; *p* < 0.05). As a group, these neurons exhibited comparable ΔF/F_0_ activity during both nonREM and REM sleep (0.81 ± 0.09 versus 0.83 ± 0.11; *p* > 0.05). However, based on the fluorescence activity during REM sleep, these neurons were further classified into two sub-categories:

*(a) Wake-max neurons:* These neurons constituted most of the wake-active neuronal type (116 of 132; 88%) encountered. The ΔF/F_0_ activity of these neurons exhibited significant changes in relation to sleep-waking state changes (Chi-square = 286.21; *p* < 0.001). These neurons, as a group, exhibited the highest ΔF/F_0_ activity during AW, which decreased significantly during QW (2.96 ± 0.22 versus 1.75 ± 0.15, *p* < 0.05), and declined further during nonREM sleep (1.75 ± 0.15 versus 0.82 ± 0.10, *p* < 0.05) and REM sleep (0.82 ± 0.10 versus 0.60 ± 0.09, *p* < 0.05), with all the neurons exhibiting a similar pattern. These neurons, as a group, exhibited the lowest ΔF/F_0_ activity during REM sleep. Based on the percentage decline in ΔF/F_0_ activity during nonREM and REM sleep compared to AW, three neurons were identified as weak (25–50%), eight as moderate (>50–100%), and all the other neurons as strong (>100%) wake-max neurons.

*(b) Wake/REMS-max neurons:* Of the 132 VGAT wake-active neurons encountered in the VLPO, 16 (12%) were identified as wake/REM-max neurons. The individual and mean ΔF/F_0_ activities of all wake/REMS-max neurons across the sleep-waking states are shown in Figure 3. There were significant effects of sleep-waking state changes on the ΔF/F_0_ activity of these neurons (Chi-square = 28.27; *p* < 0.001). These neurons, as a group, exhibited the highest ΔF/F_0_ activity during AW, which decreased significantly during QW (2.62 ± 0.58 versus 1.71 ± 0.33, *p* < 0.05), and declined further to the lowest levels during nonREM sleep (2.62 ± 0.58 versus 0.76 ± 0.17, *p* < 0.05). However, these neurons exhibited comparable Ca^2+^ fluorescence during AW and REM sleep (2.62 ± 0.58 versus 2.50 ± 0.47, NS).

Except for the significant difference in ΔF/F_0_ activity in REM sleep (2.50 ± 0.47 versus 0.60 ± 0.09, *p* < 0.05, Kruskal–Wallis one-way ANOVA on Ranks followed by Dunn’s method), both wake-max and wake/REMS-max neurons exhibited comparable Ca^2+^ dynamics during AW, QW, and nonREM sleep.

#### 2.2.3. State-Indifferent Neurons

These neurons constituted 6% (19 of 303 neurons) of all the VGAT neurons imaged in the VLPO. The spontaneous ΔF/F_0_ activity of a typical state-indifferent neuron as well as the individual and the mean ΔF/F_0_ activity of all state-indifferent neurons across the sleep-waking states are shown in Figure 2 and Figure 3. There were no significant effects of sleep-waking state changes on the ΔF/F_0_ activity of these neurons (Chi-square = 0.916; *p* > 0.05). These neurons exhibited less than 25% changes in Ca^2+^ dynamics across AW, QW, nonREM sleep, and REM sleep.

### 2.3. Ca^2+^ Dynamics of UNID VLPO Neurons during Sleep-Waking

Like VGAT neurons, the 106 UNID neurons imaged in the VLPO/extended VLPO exhibited heterogeneous sleep-waking Ca^2+^ ΔF/F_0_ activity and high variability between and within each state. Overall, the ΔF/F_0_ activity of UNID VLPO neurons changed significantly in relation to sleep-waking state changes (Chi-square = 17.11; *p* < 0.001). The mean ΔF/F_0_ activity of the recorded neurons during REM sleep (1.56 ± 0.12, range 0.01–6.21) was significantly higher than the mean ΔF/F_0_ activity during waking (1.11 ± 0.11, range 0.07–8.22) as well as during nonREM sleep (1.18 ± 0.10, range 0.10–7.43). The ΔF/F_0_ activities during waking and nonREM sleep were not significantly different (*p* > 0.05). However, based on the percent change in ΔF/F_0_ activity during nonREM sleep compared to AW and nonREM sleep versus REM sleep, these neurons were also classified into sleep-active, wake-active, and state-indifferent neurons (Table 1).

#### 2.3.1. Sleep-Active Neurons

This group represented 55% (58 of 106 neurons) of all the recorded UNID neurons in the VLPO/extended VLPO. Like VGAT, these neurons also exhibited higher ΔF/F_0_ activity during one or both phases of sleep compared to waking. Based on ΔF/F_0_activity during nonREM and REM sleep, these neurons were further categorized as nonREM sleep-active and REM sleep-active.

*a. nonREM sleep-active neurons:* These neurons constituted the majority of the sleep-active neurons (39 of 58; 67%). There were significant effects of sleep-waking state changes on the ΔF/F_0_ activity of these neurons (Chi-square = 84.07; *p* < 0.001). The mean ΔF/F_0_ activity of these neurons as a group and the individual activity profiles of all the recorded neurons across sleep-waking are shown in Figure 3. These neurons, as a group, exhibited the lowest ΔF/F_0_ activity during AW, which increased significantly during QW (*p* < 0.05). During nonREM sleep, the mean ΔF/F_0_ activity of these neurons increased further compared to AW or QW (*p* < 0.05), with all the neurons exhibiting a similar pattern (Figure 3). As a group, these neurons exhibited comparable ΔF/F_0_ activity during REM sleep (*p* > 0.05). However, based on the fluorescence activity of individual neurons during REM sleep, these neurons were also classified into two sub-categories:

(i) nonREMS/REMS-max neurons: These neurons constituted a majority of the nonREM sleep-active neurons (33 of 39; 85%). There were significant effects of sleep-waking state changes on the ΔF/F_0_ activity of these neurons (Chi-square = 81.76; *p* < 0.001). These neurons, as a group, exhibited the lowest ΔF/F_0_ activity during AW, which increased during QW (0.75 ± 0.09 versus 1.27 ± 0.15, *p* < 0.05), and increased further during nonREM sleep (1.27 ± 0.15 versus 1.67 ± 0.18, *p* < 0.05), with all the neurons exhibiting a similar pattern. During REM sleep, the Ca^2+^ ΔF/F_0_ activity increased further but was comparable (1.67 ± 0.18 versus 2.23 ± 0.24, NS). Based on the percentage increase in ΔF/F_0_ activity during nonREM sleep compared to AW, 22 neurons were identified as strong, 10 as moderate, and 1 as weak nonREMS/REMS-max neurons.

(ii) nonREMS-max neurons: Six of thirty-nine nonREM sleep-active neurons (15%) were identified as nonREMS-max neurons. These neurons, as a group, exhibited the highest ΔF/F_0_ activity during nonREM sleep compared to AW (1.34 ± 0.28 versus 0.60 ± 0.15, *p* < 0.05), QW (1.34 ± 0.28 versus 0.94 ± 0.27; *p* < 0.05), and REM sleep (1.34 ± 0.28 versus 0.60 ± 0.15; *p* < 0.05). The ΔF/F_0_ activities during AW, QW, and REM sleep were comparable.

Except for a significant difference in ΔF/F_0_ activity in REM sleep (2.23 ± 0.24 versus 0.60 ± 0.15, *p* < 0.5; one-way ANOVA followed by Dunn’s test), both nonREMS/REMS-max and nonREMS-max neurons exhibited comparable ΔF/F_0_ activity in AW, QW, and nonREM sleep (*p* > 0.05).

*b. REM sleep-active neurons:* Nineteen of fifty-eight sleep-active neurons (33%) were identified as REM sleep-max neurons. The individual and mean ΔF/F_0_ activities of these neurons as a group across the sleep-waking states are shown in Figure 3. There were significant effects of sleep-waking state changes on the ΔF/F_0_ activity of these neurons (Chi-square = 50.82; *p* < 0.001). These neurons, as a group, exhibited the highest ΔF/F_0_ activity during REM sleep (2.00 ± 0.19) compared to AW (0.3 ± 0.05, *p* < 0.05), QW (0.60 ± 0.07, *p* < 0.05), and nonREM sleep (0.77 ± 0.09, *p* < 0.05), with every neuron exhibiting a similar pattern.

Based on the percentage increase in ΔF/F_0_ activity during REM sleep compared to AW and nonREM sleep, except for one weak and one moderate, all the recorded neurons were identified as strongly REM sleep-active.

#### 2.3.2. Wake-Active Neurons

This group represented 35% (37 of 106 neurons) of all the recorded UNID VLPO neurons. These neurons exhibited the highest ΔF/F_0_ activity during AW or both AW and REM sleep compared to nonREM sleep. Based on ΔF/F_0_ activity changes during REM sleep, these neurons were also classified as wake-max and wake/REMS-max neurons.

*(a) Wake-max neurons*: These neurons constituted the majority of the wake-active neuronal group (22 of 37; 59%) encountered. The individual and the mean ΔF/F_0_ activity of all the recorded wake-max neurons across sleep-waking states are shown in Figure 3. The mean ΔF/F_0_ activity of these neurons changed significantly across the sleep-waking states (Chi-square = 59.21; *p* < 0.001). These neurons exhibited the highest ΔF/F_0_ activity during AW (1.97 ± 0.34), which decreased during QW (1.34 ± 0.27, *p* < 0.05), and declined further during nonREM sleep (0.72 ± 0.16, *p* < 0.05) and REM sleep (0.36 ± 0.08, *p* < 0.05). These neurons as a group exhibited the lowest ΔF/F_0_ activity during REM sleep.

Based on the percentage decline in ΔF/F_0_ activity during nonREM and REM sleep compared to AW, five neurons were identified as moderate (>50–100%), and all other neurons as strong (>100%) wake-max neurons.

*(b) Wake/REMS-max neurons*: These neurons constituted 41% (15 of 37) of the wake-active UNID neurons encountered. The ΔF/F_0_ activities of all the wake/REMS-max neurons and their mean as a group are shown in Figure 3. There were significant effects of sleep-waking state changes on the ΔF/F_0_ activity of these neurons (Chi-square = 27.56; *p* < 0.001). These neurons exhibited significantly (*p* < 0.05) lower ΔF/F_0_ activity during nonREM sleep (0.40 ± 0.09) compared to AW (1.24 ± 0.21), QW (0.93 ± 0.15), and REM sleep (1.19 ± 0.26). The ΔF/F_0_ activity of these neurons during AW and REM sleep were comparable.

Except for the significant difference in ΔF/F_0_ activity in REM sleep (1.19 ± 0.26 versus 0.36 ± 0.08, *p* < 0.05; Kruskal–Wallis one-way ANOVA followed by Dunn’s method), both wake-max and wake/REMS-max neurons exhibited comparable Ca^2+^ dynamics during AW, QW, and nonREM sleep (Figure 3).

#### 2.3.3. State-Indifferent Neurons

These neurons constituted 10% (11 of 106 neurons) of all the UNID neurons imaged. There were no significant effects of sleep-waking state changes on the ΔF/F_0_ activity of these neurons (Chi-square = 0.60; *p* = 0.89). These neurons exhibited less than 25% changes in Ca^2+^ dynamics across AW, QW, nonREM sleep, and REM sleep (Figure 3).

### 2.4. Comparison between Sleep-Wake Activity Profiles of VGAT and UNID Neurons

The Ca^2+^ dynamics of UNID and VGAT neurons exhibited comparable levels of heterogeneity. Various sleep-wake-related neuronal groups, identified based on changes in fluorescence across sleep-waking states, were also largely comparable. However, UNID neurons as a group exhibited significantly lower baseline waking ΔF/F_0_ activity compared to VGAT neurons (1.11 ± 0.11 versus 1.83 ± 0.11; *p* < 0.05; Mann–Whitney rank Sum test).

### 2.5. Ca^2+^ Dynamics of VLPO Neurons: Response to Homeostatic Sleep Pressure

We examined changes in the Ca^2+^ dynamics of each major functional neuronal group, i.e., sleep-active, wake-active, and state-indifferent VLPO neurons from six mice in response to changing homeostatic sleep regulatory demands to determine if these neuronal groups play any differential role in modulating homeostatic sleep. Since both VGAT and UNID neurons exhibited comparable sleep-wake activity profiles, we combined neurons from both groups for the analysis. It is well-established that 2–3 h of SD results in the building of sufficient homeostatic sleep pressure, as measured by an increasing number of sleep attempts during SD as well as an increase in NREM sleep delta power during the recovery period [8,23,30]. The animals subjected to SD in this study also exhibited progressively increasing attempts to enter sleep (not shown) and significantly increased delta power in nonREM sleep during 1 h of the recovery period (134 ± 8%, range 107 to 176%; *p* < 0.01; Wilcoxon Signed Rank test). Some neurons exhibited higher levels of instability across the three experimental paradigms and were not analyzed. The results of these recordings for each of the major functional neuronal group are summarized below.

#### 2.5.1. Response of Sleep-Active VLPO Neurons to Homeostatic Sleep Challenges

**Figure 4:**
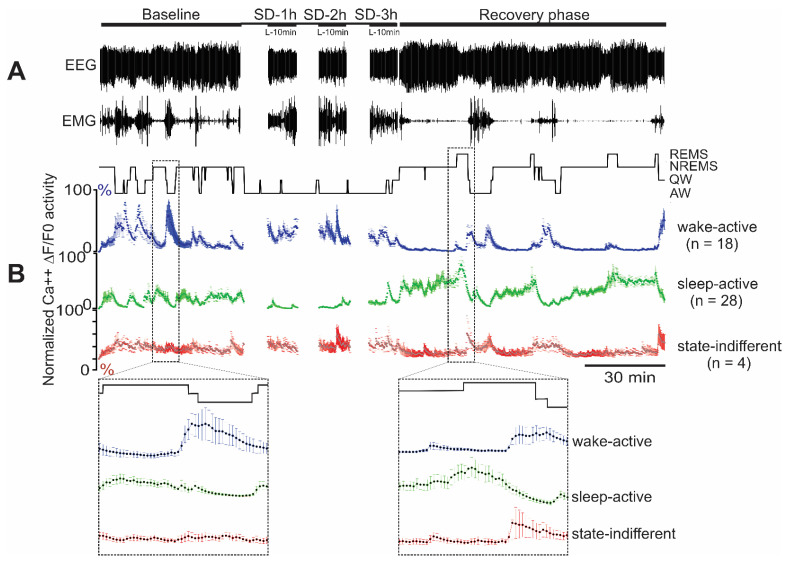
Changes in VLPO VGAT neuronal activity, as measured by levels of GCaMP6 fluorescence in response to homeostatic sleep challenges. (**A**) Traces of EEG and EMG recording along with the hypnogram showing behavioral states of the mice during baseline, during 3 h of sleep deprivation (last 10 min of each h shown), and during 1 h of the recovery period. (**B**) Colored three panels show ΔF/F_0_ (normalized mean ± SEM) of all the wake-active (*n* = 18), sleep-active (*n* = 28), and state-indifferent (*n* = 4) neurons recorded from the VLPO of this mouse during baseline, SD (last 10 min of each h), and recovery period. The marked sections during the baseline and the recovery period have been zoomed in for better visualization of the response variabilities within and among different neuronal groups.

**Figure 5:**
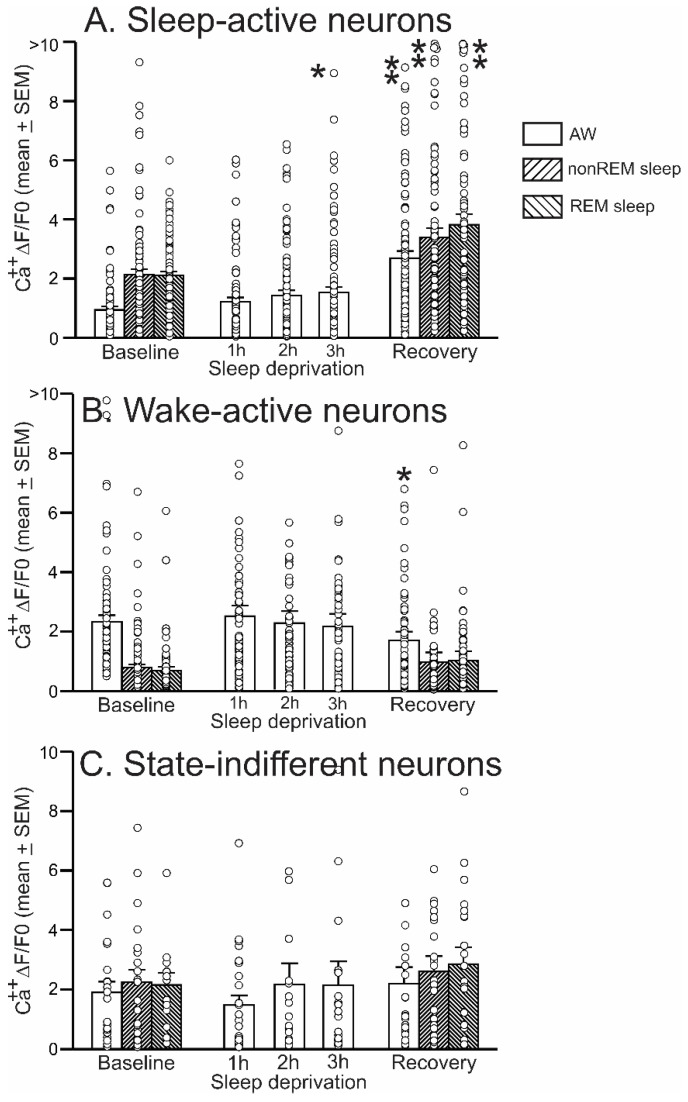
Responses of VLPO sleep-wake neuronal groups to homeostatic sleep challenges. Ca^2+^ ΔF/F_0_ of sleep-active (*n* = 91), wake-active (*n* = 84), and state-indifferent neurons (*n* = 21) as a group (mean ± SEM; bars) and those of individual neurons in each group (blank circles) during baseline, during last 10 min of each h of sleep deprivation, and during the recovery period. Compared to the baseline activity, sleep-active neurons exhibited increased activity during 2/3 h of SD and increased activity in nonREM sleep and REM sleep during the recovery period. Compared to baseline, wake-active neurons did not exhibit significant changes during SD but showed a decline in waking activity during the recovery period. State-indifferent neurons were largely non-responsive to SD. *, *p* < 0.05; **, *p* < 0.01 levels of significance, Friedman RM ANOVA on Ranks followed by Student–Newman–Keuls test; Wilcoxon Signed Rank test).

The Ca^2+^ ΔF/F_0_ activity of 91 sleep-active neurons was quantified during baseline, 3 h of SD, and 1 h of the recovery period. An example of continuous recording of Ca^2+^ ΔF/F_0_ activity of all sleep-active VLPO neurons encountered in one representative mouse across the three conditions, i.e., spontaneous sleep-wake activity during the baseline period, waking activity during 10 min at the end of each h of SD, and sleep-wake activity during ~1 h of the recovery period, is shown in Figure 4. Additionally, the mean ΔF/F_0_ activity of sleep-active neurons as a group, along with the activity profiles of all individual neurons encountered, is shown in Figure 5.

The ΔF/F_0_ activity of VLPO sleep-active neurons exhibited significant changes in its dynamics in response to SD (Chi-square = 127.15; *p* < 0.001). During the baseline period, sleep-active neurons exhibited elevated mean ΔF/F_0_ activity during nonREM and REM sleep compared with waking (Figure 4 and Figure 5). At the start of SD, only infrequent interventions were required to keep the animal awake, and the ΔF/F_0_ activity of these neurons remained comparable to the baseline activity. As homeostatic sleep pressure increased with continuing SD, the ΔF/F_0_ activity of these neurons increased and became significantly higher compared to the baseline activity during the third hour of SD. The waking ΔF/F_0_ activity of these neurons remained elevated during the recovery period (*p* < 0.01). These neurons also exhibited significantly higher ΔF/F_0_ activity during nonREM sleep (*p* < 0.01) and REM sleep (*p* < 0.01), compared to their respective baseline activities. The nonREM sleep during the recovery period was also marked by increased delta activity compared to the baseline nonREM sleep (134 ± 8%, *p* < 0.01).

#### 2.5.2. Response of Wake-Active VLPO Neurons to Homeostatic Challenge

The Ca^2+^ ΔF/F_0_ activity of 84 wake-active VLPO neurons was quantified during baseline, SD, and recovery. An example of the fluorescence activity of the wake-active neurons encountered in one mouse across the three conditions as well as the ΔF/F_0_ activity profiles of all wake-active neurons examined from six mice and their mean are shown in Figure 4 and Figure 5, respectively.

The ΔF/F_0_ activity of VLPO wake-active neurons exhibited significant but marginal changes in its dynamics in response to SD (Chi-square = 26.60; *p* < 0.001). During the baseline, as a group, the wake-active neurons exhibited the highest ΔF/F_0_activity during waking and the lowest activity during nonREM and REM sleep. During SD, ΔF/F_0_activity of wake-active neurons did not exhibit any significant change and remained comparable to the activity during the baseline condition (Figure 5). However, the waking ΔF/F_0_activity of these neurons exhibited a significant decline (*p* < 0.05) during the recovery period. These neurons also showed comparable ΔF/F_0_ activity in nonREM and REM sleep during the recovery period, compared to their respective baseline activities (Figure 5).

#### 2.5.3. Response of State-Indifferent VLPO Neurons to Homeostatic Challenge

The Ca^2+^ ΔF/F_0_activity of 21 state-indifferent VLPO neurons was quantified during baseline, SD, and recovery. During the baseline, these neurons, as a group, exhibited comparable ΔF/F_0_activity across the sleep-wake stages. With increasing sleep pressure during SD, the ΔF/F_0_activity of state-indifferent neurons did not exhibit any significant changes compared to their baseline activity (Chi-square = 5.92; *p* = 0.21; Figure 5). The ΔF/F_0_ activity of these neurons in waking, nonREM, and REM sleep during the recovery period was also comparable to their baseline activity (Figure 5).

## 3. Discussion

The VLPO contains GABAergic/galaninergic sleep-active neurons that are hypothesized to play critical sleep regulatory functions. However, the extent to which VLPO GABAergic neurons are activated in response to the expression of spontaneous sleep and to homeostatically driven sleep regulatory demands is not fully understood. In this study, using a miniature single-photon epifluorescence microscope, we measured the changes in Ca^2+^-induced fluorescence in GCaMP6 expressing VGAT and UNID VLPO neurons across spontaneous sleep-waking and in response to the waxing and waning of homeostatic sleep drives. We found that the majority of both VGAT and UNID VLPO neurons were sleep-active (VGAT neurons, 50%; UNID neurons, 55%). These sleep-active neurons also seemed to sense homeostatic sleep challenges, since their activities reflected changes in homeostatic sleep pressure in animals subjected to short-term SD. These neurons started exhibiting increased activity during waking after 2 h of SD (in parallel with increasing homeostatic sleep pressure), which remained elevated during the recovery period. These neurons also exhibited increased fluorescent activity in nonREM sleep, which was marked by elevated EEG SWA, an electrophysiological biomarker of homeostatic sleep pressure, and in REM sleep during the recovery period. On the other hand, wake-active neurons only exhibited a decline in activity during the recovery period. State-indifferent neurons did not show significant changes in Ca^2+^ dynamics in response to spontaneous or homeostatic sleep demands.

Generally, in vivo electrophysiology, especially extracellular unit recordings, has been used to characterize the sleep-wake activity profiles of neurons in various brain regions, including those in the VLPO [8,9,31,32,33,34,35]. In recent years, Ca^2+^ imaging, which uses changes in intracellular levels of Ca^2+^ associated with neuronal depolarization as a readout of neuronal activity levels, has been used to more precisely dissect the role of specific neural groups and circuitry in particular function/behavior, or validate findings of those existing in vivo electrophysiological and immunohistochemical studies [27,29,36,37].

To our knowledge, this is the first study that has used changes in Ca^2+^ dynamics to fully characterize the activity profiles of VGAT or GABAergic vis-a-vis UNID VLPO neurons across spontaneous sleep-waking and in response to homeostatic sleep regulatory challenges in freely behaving mice. Based on the nonREMS/AW or nonREMS/REMS Ca^2+^ fluorescent activity, we found that while the sampled VGAT neurons exhibited heterogeneous sleep-wake activity profiles, the majority of them (152 of 303 neurons; 50%) exhibited increased activity in one or both phases of sleep compared to waking. This population included neurons that were more active during both nonREM sleep and REM sleep (nonREMS/REMS-max; 96 of 120 neurons) compared to waking and those that were relatively more active during nonREM sleep (nonREMS-max; 24 of 120 neurons). A subpopulation of sleep-active neurons was selectively more active in REM sleep (REMS-max; 32 of 120 neurons). On the other hand, 132 of 303 (44%) VGAT neurons were identified as wake-active, including wake-max (116 of 132) and wake/REMS-max neurons (16 of 132), and 19 of 303 (6%) were identified as state-indifferent neurons. Among the UNID VLPO neurons, comparable populations of sleep-wake-related phenotypes were also identified (sleep-active, 58 of 106, 55%; wake-active, 37 of 106, 35%; and state-indifferent, 11 of 106, 10%). The minor differences in the percentage of various neuronal groups among VGAT and UNID neurons could be due to a relatively small sample size of UNID neurons (106 neurons from three mice) and differences in the dorso-ventral and medial-lateral localization of the GRIN lenses. Since the percentage of UNID neurons that were sleep-active is comparable to VGAT sleep-active neurons, it is likely that most of the UNID sleep-active neurons sampled in this study were also GABAergic and became transfected with GCaMP6.

Based on reconstructions of the GRIN lens’s locations, all 303 VGAT and 106 UNID neurons described in this study were localized in the LPO, including in the VLPO and to some extent in the DLPO and adjoining areas (Figure 1). These sleep-active VGAT and UNID neurons were intermingled with other sleep-wake neuronal types in these regions. Our findings are consistent with earlier unit recording and double-label immunohistochemical studies suggesting that while sleep-active neurons, including GABAergic/galaninergic neurons exhibiting sleep-associated Fos-IR, are distributed throughout the POA, a relatively higher concentration of such neurons is localized in the VLPO and adjoining areas [8,9,16,23,32]. The prevalence of VGAT or UNID sleep-active neurons in the VLPO is consistent with their role in regulating one or more components of sleep.

In this study, various sleep/wake neuronal groups among the VGAT and UNID neurons were categorized solely on the basis of nonREMS, REMS/AW, or nonREMS/REMS ΔF/F_0_ activity ratios. Extracellular unit recording studies from VLPO in rats and the preoptic region in mice have also identified similar sleep-wake neuronal phenotypes in largely comparable populations (Ca^2+^ imaging in this study versus unit recording; sleep-active neurons, 50–55% vs. 49% in mice and 43–56% in rats; wake-active neurons, 35–44% versus 41% in mice and 37–49% in rats; state-indifferent neurons, 6–10% versus 11% in mice and 19–24% in rats). Additionally, similar to the extracellular discharge activity studies, the majority of VGAT and UNID sleep-active neurons in this study were identified as strongly sleep-active [8]. The minor differences in the population of sleep-wake-related neuronal groups could be due to the differences in the criteria of classification used and differences in the localization of GRIN lenses versus microwires (or the sub-region/area sampled) used in these studies. Additionally, unlike unit recordings, where changes in extracellular discharge activity were fast and could be easily assessed with reference to rapidly changing EEG synchronization and desynchronization or during 5–10 s transitions to EEG-defined nonREM sleep onset, changes in calcium dynamics seemed slower. Overall, the presence of nonREMS-max, nonREMS/REMS-max, and REMS-max neurons in the VLPO supports its role in sleep regulation.

Immunohistochemical studies suggest that most of the sleep-active neurons in the VLPO contain the inhibitory neurotransmitter GABA and or galanin, and virtually all of the galanin neurons in the VLPO are GAD+ [13,14]. In this study, the sleep-wake activity profiles of VGAT neurons suggest that GABAergic neurons in the VLPO/extended VLPO are functionally diverse, and ~50% of GABAergic neurons are not sleep-active. Thus, it is not surprising that in some studies, the activation of VLPO or POA GABAergic neurons has been reported to produce only marginal or no effects on sleep [18] Some evidence suggests that VLPO contains both local GABAergic interneurons that instead inhibit sleep-promoting neurons and GABAergic neurons projecting to critical wake-regulatory systems in the hypothalamus, including the PF-HA containing hypocretin/orexin (HCRT) and glutamatergic neurons, the tuberomammillary nucleus (TMN) containing histaminergic neurons, and brainstem monoaminergic neurons that are predominantly sleep-active and are inhibited by monoamines [4,11,13,16,20,21,38,39]. Recently, we found that even the unilateral activation of VLPO neurons projecting to the hypocretin field increased nonREM sleep during the dark phase, when rats are typically awake, and after exposure to the novel environment during the light phase, which induces acute arousal [12]. Although our approach did not allow us to assess the Ca^2+^ dynamics of these two subpopulations of VLPO GABAergic neurons, it is plausible that the majority of wake-active GABAergic neurons encountered in this study were local interneurons. The validation of such interpretations requires further studies.

One unexpected finding of this study is that a comparable population of sleep-active neurons was identified among a much more heterogeneous UNID neuronal group, given that GCaMP6 indiscriminately transfected all cell types, including wake-active glutamatergic neurons in the VLPO [40]. This suggests that while it is likely that the sleep-active UNID neurons included most of the sleep-active VGAT neurons, some of the recorded sleep-active UNID neurons could be non-GABAergic. Additionally, UNID VLPO neurons exhibited low fluorescence compared to VGAT neurons. We used V3 and V4 versions of the miniscope for Ca^2+^ imaging of UNID and VGAT neurons, respectively. The difference in fluorescence levels could be due to the different versions of the miniscopes used. Although our classification of sleep-wake relatedness of neurons is based on relative fluorescence during AW, nonREMS, and REMS of each neuron, the possibility that a relatively low sensitivity of the V3 miniscope or differential Ca^2+^ dynamics of GABA and glutamatergic neurons may have contributed to the detection of relatively fewer wake-active neurons among the UNID neuronal population cannot be completely ruled out.

The findings of the present study suggest that while the activation of VLPO sleep-active neurons is strongly reflective of sleep expression, these neurons also respond to changing homeostatic sleep pressure. These neurons started exhibiting increased activity during waking itself after 2/3 h of SD, apparently with increasing homeostatic sleep pressure that persisted during the early recovery period. These neurons also exhibited increased activity in nonREMS, which was also marked by elevated EEG SWA, an electrophysiological biomarker of homeostatic sleep pressure, and in REM sleep during the recovery period. On the other hand, wake-active neurons exhibited a decline in activity only during recovery after SD, which potentially reflects increased inhibitory influences on these neurons due to the activation of sleep-active neurons and increased homeostatic sleep pressure. These findings on the temporal relationships between the dynamics of VLPO sleep-active neurons and homeostatic sleep pressure are largely consistent with the findings of an earlier unit recording study in rats and the role of VLPO in homeostatic control of sleep [8].

## 4. Materials and Methods

### 4.1. Experimental Subjects

Experiments were conducted on four wild C57BL6 (Charles River) and eight VGAT-IRES-Cre knock-in male and female mice that were 5–8 months of age and weighed 28–35 g at the time of data acquisition. The VGAT-IRES-Cre::L10 breeding pairs were provided by Dr. V. Ramalingam, Beth Israel Deaconess Medical Center, Harvard Medical School. These mice were bred in our Veterinary Medical Unit, and those found to be VGAT-IRES-Cre (genotyping performed by Transnetyx, Inc., Cordova, TN, USA) were selected for Ca^2+^ imaging. However, data from 1 WT and 1 VGAT-IRES-Cre mouse could not be used due to mechanical failure. The mice were maintained on a 12 h:12 h light/dark cycle (lights on at 8:00 h), an ambient temperature of 24 ± 2 °C, and with food and water available ad libitum. All experiments were conducted in accordance with the National Research Council’s “Guide for the Care and Use of Laboratory Animals” and were approved by the Institutional Animal Research Committee of the Veterans Affairs Greater Los Angeles Healthcare System.

### 4.2. Surgical Procedures

The details of the general surgical procedures have been described previously [12,41]. The preparation of mice for Ca^2+^ imaging of VLPO neurons was performed in two steps.

*Viral vector injection:* First, under surgical anesthesia (100 mg/Kg ketamine + 15 mg/Kg xylazine; maintenance with isoflurane) and aseptic conditions, mice were placed in a stereotaxic frame, the skull was exposed and cleaned, and a hole (~1 mm diameter) was drilled unilaterally in the skull just above the VLPO. Then, the needle (28 G) of a micro-syringe (Hamilton Co., Reno, NV, USA) loaded with viral vector and attached to a motor-driven micromanipulator was inserted through the hole into the VLPO (AP: 0.1 mm, LM: 0.5 mm from bregma, and DV: 5.6 mm from the scalp) [12,42] for delivering viral vector. C57BL6 and VGAT-IRES-Cre mice were injected with 0.2–0.5 µL (titer ≥ 1 × 10^13^ vg/mL) of AAV-Syn-GCaMP6-WPRE-SV40 and AAV-EF1a-DIO-GCaMP6-P2A-nls-dTomato (#100837-AAV1 and, #51082-AAV1; AddGene), respectively, into the VLPO. The viral vectors were injected slowly over a 10–25 min period (0.1 µL/5 min), and after injection, the needle was left in place for another 20 min and then slowly withdrawn to avoid any backflow. After viral injections, the open wounds were sutured, and mice were returned to their home cages for 3–4 weeks, which was sufficient for expressing viral genes before the implantation of gradient index (GRIN) lens and electroencephalogram (EEG) and electromyogram (EMG) electrodes. AAV-Syn-GCaMP6-WPRE-SV40 and AAV-EF1a-DIO-GCaMP6-P2A-nls-dTomato injections into the VLPO resulted in GCaMP6 expression in a large number of neurons in WT- and Cre-dependent expression of GCaMP6 exclusively in VGAT neurons, as evident from green, fluorescent labeling (Figure 1) in VGAT-IRES-Cre mice.

*Implantation of EEG/EMG electrodes and GRIN lens*: EEG and EMG electrodes and GRIN lenses were implanted after 3–4 weeks of viral injections, using the same general surgical procedure. Again, under surgical anesthesia and aseptic conditions, the mouse was placed in a stereotaxic frame, and the skull was exposed and thoroughly cleaned of connective tissue. Then, using the same stereotaxic coordinates, one ~2 mm-diameter hole was drilled on the ipsilateral side directly above the injection site/VLPO for the implantation of the GRIN lens, and two holes (~0.6 mm in diameter) were drilled on the contralateral side for the implantation of EEG electrodes. EEG electrodes were screwed onto the skull, and flexible EMG wire electrodes were implanted into the dorsal neck muscle. At this time, a GRIN lens (0.5 mm diameter; 8.4 mm length; Inscopix, IPN: 130-000152) was implanted over the VLPO/viral injection site (target location; AP: 0.1 mm, LM: 0.5 mm, and DV: 5.4–5.5) using a custom-made holder attached to a stereotaxic manipulator. Once in place, the lens was secured to the skull using superglue and dental cement and covered with a miniature plastic cap to protect the lens surface. At this time, the connector with EEG and EMG electrodes was also fixed to the skull with dental cement. After surgical implantations of EEG and EMG electrodes and GRIN lenses, mice were returned to their home cages.

### 4.3. Post-Surgical Recovery and Adaptation

Mice were allowed to recover from the surgical procedure for 2–4 weeks in Plexiglas recording cages with free access to food and water and placed in a sound-attenuated and temperature-controlled recording chamber maintained at the same 12-h:12 h light/dark cycle. During the recovery phase, mice were observed for gross behavioral and sleep-wake abnormalities. Then, the miniscope baseplate was mounted onto the mouse head under visual guidance using the attached miniscope to determine the best field of view. The lens implantation and base-plating were performed according to the tutorial video (https://www.youtube.com/watch?v=SZPAQps_uVo, accessed on 1 November 2021; https://www.youtube.com/watch? v=z_32O2XoYI4&t=2s, accessed on 1 November 2021), detailed instructions on the website (miniscope.org/index.php/Surgery Protocol, Nov-Dec 2021), and earlier studies [43,44]. One week after base-plating, mice were connected to the EEG/EMG recording cables and miniscope and allowed 3–4 h/day of further acclimatization to the recording cables and miniscope for 3–4 days. The quality of EEG/EMG and Ca^2+^ signals was checked at this time. During this time, mice were also adapted to handling similar to what they were subjected to during the sleep deprivation procedure. The operated mice exhibited a typical sleep-wake pattern.

### 4.4. Miniscope and Software

We used v3 and v4 miniscopes on wild-type and VGAT-IRES-Cre mice, respectively. Miniscopes were assembled in our lab from parts obtained from multiple suppliers (For details, see website; http://miniscope.org/index.php/Main_Page, accessed on 4 April 2023). While we used multiple miniscopes, the same device was used across the experiments in each mouse to avoid signal variabilities. Drivers and acquisition software were downloaded from open sources (GitHub https://github.com/daharoni/Miniscope_DAQ_Firmware and https://github.com/daharoni/Miniscope_DAQ_Software (accessed on 4 April 2023)) and used according to detailed instructions from the website (http://miniscope.org/index.php/Main_Page, accessed on 4 April 2023).

### 4.5. Sleep-Waking and Ca^2+^ Signal Recording

EEG, EMG, and cytoplasmic Ca^2+^-induced fluorescence were simultaneously recorded during the light phase (recording session typically started in the middle of the light phase) with mice in their home cages placed in the sound-attenuating chamber. EEG and EMG signals were amplified, digitized, and stored on the hard disk of the host computer (Cambridge Electronic Design 1401; supporting software, Spike 2) for subsequent sleep-wake scoring and analyses [30,41]. Ca^2+^ signals were acquired by the miniscope at 10 frame/s and stored as multiple consecutive video files in .avi format on the hard disc. Recordings of EEG/EMG and Ca^2+^ signals were started manually: it began with Ca^2+^ followed by EEG/EMG, causing a delay of 1–3 s in data acquisition, which was aligned during analysis.

Ca^2+^ dynamics of VLPO neurons across spontaneous sleep-waking: During the first recording session, EEG and EMG signals and Ca^2+^ imaging data were acquired at least across three spontaneous sleep-waking cycles, which typically took 45 min–1 h (Figure 1).

Ca^2+^ dynamics of VLPO neurons in response to changing homeostatic sleep regulatory demands: In the second session, changes in sleep-wake architecture and the Ca^2+^ signals from VLPO neurons were recorded during the waxing and waning of the homeostatic sleep drive. This recording session started after at least one week to compensate for any fading of the calcium indicator during the first session. EEG and EMG signals were recorded continuously across three sleep-waking cycles as a baseline, then during the 3 h of SD by “gentle handling” [30], and followed by 1 h of the recovery sleep (RS) period. In a few cases, the session started with SD, followed by the RS period. In those cases, data obtained during the baseline recording day were used for comparison. While Ca^2+^ signal was continuously recorded during the baseline and recovery period, during SD, to avoid fading of the fluorescence intensity of the calcium indicator, it was recorded only for 10 min at the end of each h of SD (Figure 1).

### 4.6. Tissue Processing and Localization of the Recording Sites

At the end of the recording session, mice were deeply anesthetized (100 mg/kg, IP, pentobarbital) and perfused transcardially with phosphate-buffered saline (PBS, 0.1 M, pH 7.2), followed by 4% paraformaldehyde in PBS [30,41]. Brains were extracted, placed into 30% sucrose solution until they sank, and cut coronally at 40 µm using a freezing microtome. Sections were mounted and cover-slipped using a fluorescence mounting medium (ProLong™ Gold Antifade Mountant, P36931, ThermoFisher Scientific). The GRIN lens track and the area below were identified, and fluorescent images, including GCaMP6+ and dTomato+ (not shown) neurons under the bottom part of the track, were acquired using an LSM 900 Zeiss confocal microscope.

### 4.7. Data Analysis

Sleep-wake scoring and extraction of nonREM sleep SWA: The EEG/EMG and imaging data files were coded so that data could be processed and analyzed in a blind manner. Sleep-wake profiles of animals were scored manually in 10 s epochs in terms of waking (consisting of active, AW, and quiet-waking, QW), nonREM sleep, and REM sleep using standard criteria [30,41]. EEG delta power (2–4 Hz) was calculated for each epoch of nonREM sleep encountered during the entire recording session and then averaged for baseline and during the first hour of the recovery period. Scoring and delta extraction were performed using Spike2 software.

Ca^2+^ image processing: Video files of Ca^2+^ imaging signals after concatenation, cropping, and processing in ImageJ were further processed for motion correction and signal extraction using Python-based open source software CaImAn and MiniAn (doi.org/10.7554/eLife.38173, doi.org/10.7554/eLife.70661) downloaded from GitHub. To run Python, additional open-source software Anaconda (anaconda.org) and Jupyter Notebook (jupyter.org) were used.

To further improve motion correction, the video was cropped into multiple smaller parts. Each part had the same length as the original video but included 3–20 neurons and was processed separately for signal extraction. Neurons on video were identified as units, and Ca^2+^ signal of each unit was extracted, and their ΔF/F_0_ expressed as z-score traces and saved as .csv files. In addition to automatic assessment, the quality of ΔF/F_0_signal of each unit was assessed manually and removed from analysis if the average fluorescent intensity of the unit across sleep-waking states was <0.5. For aligning the timeline of unit tracing and the somnogram, the ΔF/F_0_data were down-sampled by averaging 10 s of ΔF/F_0_signal to correspond to the 10 s epoch of EEG and EMG scoring.

Classification of neurons: To determine sleep-wake activity profiles of neurons, we averaged *Z*-scored ΔF/F_0_ of each neuron across all the episodes of AW, QW, nonREM sleep, and REM sleep encountered during the entire baseline or recovery recording session in the case of SD. We believe that such analyses provide a better and unbiased assessment of sleep-wake activity profiles of the neurons than profiling based on their activity during a limited number (typically 2–4 episodes) and selected episodes of each state. In SD study, in three mice, baseline, SD, and RS data were acquired on the same day. In three mice, however, the baseline sleep-wake imaging, especially in REM sleep, could not be obtained within a comparable timeframe, and thus, the data obtained on day 1 was used as the baseline.

A minimum of ≥25% change in ΔF/F_0_ activity between sleep-waking states was used as a criterion for neuronal classification in relation to sleep-waking behavior. Each classification iteration was validated with logistic regression (LR), as recommended for continuous explanatory variables and binary categorical outcome variables [45]. The logistic regression calculations were performed using the statsmodels library for Python programming language. Neurons were classified as “wake-active” if their nonREMS/AW ΔF/F_0_ activity ratios were <0.75 (LR coef. = −1.26, *p* < 0.001); wake/REM-max, if nonREMS/AW ratio was <0.75 (LR coef. = −12.64, *p* < 0.001) and REM/AW ratio was >0.75 and <2.0 (LR coef. = 5.60, *p* < 0.001); nonREM sleep-active, if nonREM/AW ratio was >1.25 (LR coef. = 0.19, *p* < 0.001); nonREMS/REMS-max were identified among nonREM sleep-active neurons if nonREMS/AW ratio was >1.25 and nonREMS/REMS ratio was >0.5 (LR coef. = 2.72, *p* < 0.001); REMS-active if REM/AW (LR coef. = 0.017, *p* < 0.001) and REM/nonREMS (LR coef. = −0.287, *p* < 0.001) ΔF/F_0_ activity ratios were >2.0. All remaining neurons exhibiting <25% change in ΔF/F_0_ activity across sleep-waking states were classified as state-indifferent neurons.

Statistical analyses: The SigmaPlot (Systat Software, San Jose, CA, USA) software package was used for statistical analyses of the data. Data are presented as mean ΔF/F_0_ activity ± standard error of the mean (SEM). In the majority of cases, the data failed normality and or equal variance tests, and thus nonparametric tests were used for all the statistical analyses. Friedman RM ANOVA on Ranks followed by Student–Newman–Keuls method was used to compare changes in ΔF/F_0_activity across AW, QW, nonREM, and REM sleep or for comparing activity during baseline, SD, and recovery sleep. Changes in ΔF/F_0_ activity in nonREM sleep, and REM sleep during the baseline were compared to ΔF/F_0_ activity during the recovery period after SD using the Wilcoxon Signed Rank test. Activity profiles of various neuronal groups were compared (between group comparisons) using Mann–Whitney Rank Sum test or Kruskal–Wallis one-way ANOVA on Ranks, followed by Dunn’s method.

## 5. Conclusions

The findings of the present study suggest (i) that the activation of VLPO sleep-active neurons, including GABAergic neurons, is strongly reflective of sleep expression, and (ii) that VLPO sleep-active neurons respond to changing homeostatic sleep regulatory demands. These neurons start exhibiting increased activity after a certain threshold of homeostatic sleep pressure during sleep deprivation that persists during nonREMS with elevated EEG SWA and in REM sleep during the recovery period. The activity profiles of sleep-active neurons in the VLPO, as quantified by calcium imaging, further support the VLPO’s role in regulating one or more components of sleep.

## Figures and Tables

**Table 1:** Sleep-wake activity profiles of VLPO neurons recorded in each mouse.

Animal ID	Sex	NREMS-Active Neurons		Wake-Active Neurons		State-Ind	Total
		NREMS-Active	REMS-Active	W-Max	W/REMS-Max	Neurons	
**VGAT neurons**							
VGAT #7	M	7	5	7	3	1	23
VGAT#8	M	11		8		2	21
VGAT#11	F	21	13	32	3	2	71
VGAT#12	F	15	3	6	1	1	26
VGAT#14	M	21	1	22	5	3	52
VGAT#15	F	13	9	29	2	6	59
VGAT#16	M	32	1	12	2	4	51
**Total**		**120**	**32**	**116**	**16**	**19**	**303**
**Unidentified neurons**							
#17	M	22	8	7	2	7	46
#1	F	5	6	13	3	2	29
#24	M	12	5	2	10	2	31
**Total**		**39**	**19**	**22**	**15**	**11**	**106**

## Data Availability

The result section sufficiently describes the data. Further details or the raw data or analysis performed are available for sharing/presentation upon request to the corresponding author.
